# The effect of trans-theoretical model stage-matched intervention on medication adherence in hypertensive patients

**DOI:** 10.3389/fcvm.2024.1470666

**Published:** 2024-11-28

**Authors:** Kamran Saeidi, Tina Ghavami, Soodeh Shahsavari, Soraya Siabani, Fatemeh Rajati

**Affiliations:** ^1^ Department of Health Education and Health Promotion, School of Public Health, Kermanshah University of Medical Sciences, Kermanshah, Iran; ^2^ Student Research Committee, Kermanshah University of Medical Sciences, Kermanshah, Iran; ^3^ Department of Health Information Technology, School of Paramedical Sciences, Kermanshah University of Medical Sciences, Kermanshah, Iran

**Keywords:** hypertension, blood pressure, stages of change, Transtheoretical Model, health education, adherence, lifestyle, physical activity

## Abstract

**Introduction:**

Hypertension is a chronic condition that requires active patient management and awareness of treatment strategies. This study aimed to evaluate the effectiveness of an intervention program grounded in the Transtheoretical Model (TTM) of behavior change for improving treatment adherence among hypertensive patients.

**Materials and methods:**

This study conducted at the Nukan Comprehensive Rural Health Center in Kermanshah, Iran, 120 participants were selected according to specific inclusion criteria. Demographic data and responses to 20 hypertension-related behavior questions were collected via a questionnaire. Participants were categorized into non-adherence (pre-contemplation, contemplation, preparation stages) and adherence categories (action and maintenance stages) based on self-reported medication adherence, with 60 individuals in each group. Each group was then randomly divided into intervention and control subgroups. The educational intervention consisted of four 45 min sessions grounded in TTM constructs regarding to health-related behaviors including Physical activity, salt and oil intake, and fruit and vegetable consumption, and medication adherence. Three months post-intervention, a follow-up questionnaire evaluated the educational impact on treatment adherence. The McNemar test and Chi-square test were utilized to analyze effects across the intervention, control, and pre- and post-intervention groups.

**Results:**

The participants had a mean age of 58.09 years (SD = 11.85). Three months after the intervention, the non-adherence intervention group showed significant progress in transitioning to the action and maintenance stages across all physical activity behaviors, as well as in salt, oil, fruit and vegetable intake, and medication adherence (*P* < 0.005). In the adherence intervention group, after the intervention, the number of hypertensive patients who fell into the action and maintenance categories according to all lifestyle variables increased, but the change was not significant. Concerning blood pressure, the intervention group had a significant reduction in mean systolic blood pressure (142.88 ± 20.87 vs. 141.00 ± 18.52; *p* = 0.015), but the decrease in mean diastolic blood pressure was not significant (88.17 ± 10.30 vs. 87.58 ± 9.70; *p* = 0.154). No significant changes in systolic or diastolic blood pressure were observed in the control or in intervention groups within the adherence category.

**Conclusion:**

This research highlights the potential benefits of applying the TTM to tailor interventions for hypertensive patients with poor treatment adherence, suggesting that such an approach can enhance the efficacy of health education interventions.

## 1 Introduction

Globally, hypertension is estimated to affect 33% of adults aged 30–79, and it is projected that there will be over 500 million individuals with hypertension, constituting approximately 60% of the global population by 2025 (1, 2). The standardized prevalence of hypertension, according to the JNC7 guideline, was estimated to be 22.3% (95% CI: 20.6–24.1) among Iranian population from 2014 to 2020 (3). In the Kurdish population of Iran, the prevalence of hypertension is estimated to be 15.7% (4). Hypertension is recognized as a significant controllable factor for cardiovascular diseases (CVDs), causing more fatalities than any other risk factor in Asian areas. This risk factor is largely preventable and controllable, which means that taking appropriate preventive and therapeutic measures can prevent its occurrence and development (1).

Adherence to the therapeutic regimen among patients with cardiovascular and hypertensive diseases necessitates compliance with both pharmacological and non-pharmacological methods (5). Insufficient adherence can result in elevated treatment costs and negative health outcomes (6). Traditionally, educational interventions for hypertension management have not taken into account the stage of behavioral change and the readiness of patients to engage in that change. Most interventions, based on health education models, typically focus on specific constructs without addressing the corresponding stage of change. This approach creates a gap in effectively aligning the type of intervention with the individual's behavioral readiness. The Transtheoretical Model (TTM) is a dynamic and continuous framework for behavioral change, designed to address the varying needs of patients at different stages of change. It categorizes individuals into specific stages, allowing for tailored interventions based on their current stage of change (7).

The TTM has widespread application in behavioral research, primary and secondary prevention, and has been used for some unhealthy behaviors such as smoking, obesity, substance use, and hypertension. This model is an integrative model of voluntary change that covers the stages of change (8). This model consists of four main elements, including stages of change, processes of change, decisional balance, and self-efficacy. The stages of change categorize an individual's readiness for change into five distinct categories: precontemplation, contemplation, preparation, action, and maintenance (7). Previous studies highlight the effectiveness of stage-matched lifestyle management for patients with type 2 diabetes, demonstrating significant improvements in health outcomes (9). However, a systematic review indicated that limited studies have considered the stage of change for patients with hypertension (10). Furthermore, none of these studies accounted for adherence to medication as a criterion for categorizing hypertension patients into non-adherence category (precontemplation, contemplation, or preparation stages) or adherence category (action or maintenance stages).

In this study, we aim to investigate the impact of an educational program designed according to the stages of behavioral change outlined in the Transtheoretical Model on the adherence levels to self-management behaviors among patients with hypertension.

## 2 Materials and methods

### 2.1 Design and study population

This research was an interventional study conducted based on the TTM of behavior change. The study population consisted of 120 patients with high blood pressure who were selected from the Nukan Comprehensive Rural Health Service Center in Kermanshah city in December 2019. Participants were selected using a simple random sampling method from the medical records of patients referred to health houses who were diagnosed with hypertension by a physician. The inclusion criteria were patients with hypertension aged 30 years or older, and whose hypertension had been previously confirmed. The exclusion criteria were patients with mental illness, cognitive-social disorders, and pregnant women with hypertension. They were initially examined by a general physician, and none were diagnosed with mental illness or cognitive-social disorders.

As simple randomization may introduce confounding variables affecting internal validity. Potential confounders include age, comorbidities, and baseline medication adherence. We controlled for these variables through stratification.

**Figure 1:**
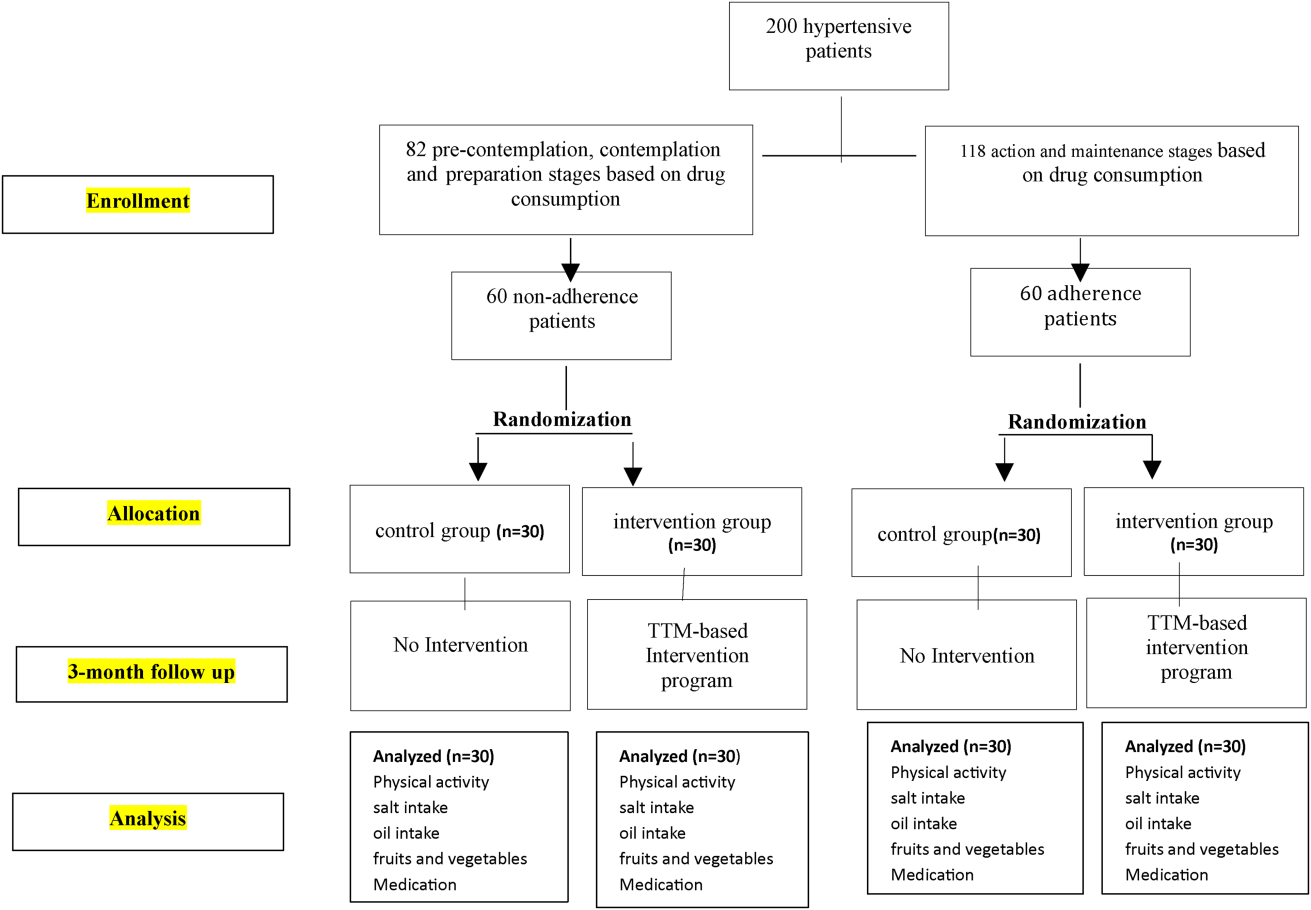
Flowchart of participant selection for the intervention and control groups.

In this study, considering that medication use is the most effective factor in controlling hypertension (11), individuals who were in the pre-contemplation, contemplation, and preparation stages of medication use were classified into the non-adherence category, and those who were in the action and maintenance stages were classified into the treatment adherence category. Then, each of the two groups was randomly divided into control and intervention subgroups (Figure 1).

The sample size was estimated to be at least 17 participants in each group, using the formula for the difference in means, with *α* = 0.05 and *β* = 0.20, as reported in the study by Sadeghi et al. (12).

$$n_{1}=\frac{2\sigma^{2}{\left(z_{1-\frac{\alpha }{2}}+z_{1-\beta }\right)}^{2}}{{\left(\mu_{2}-\mu_{1}\right)}^{2}}$$

$\mu_{1}$ = The difference in the mean diastolic blood pressure before and after the intervention in the control group.

$\mu_{2}$ = The difference in the mean diastolic blood pressure before and after the intervention in the experimental group.

$\sigma^{2}$ = The variance: 16.48.

α = The type I error: 0.0 5.

In this study, to ensure sufficient statistical power and establish the normality assumption within each group, a sample size of 30 participants was considered. The sample size of 120 was determined through a power analysis, which indicated that this number would provide sufficient power to detect significant differences based on expected effect sizes from previous studies.

### 2.2 Measurements

The measurement tool included a questionnaire, with the first section collecting demographic information and the second section comprising 20 questions related to hypertension-associated behaviors. The demographic information section consisted of 14 questions covering gender, age, height, weight, waist circumference, marital status, employment status, educational level, income, comorbidities, duration of hypertension, current and past smoking status, and hypertension medication use. The second section was designed with a total of 20 questions, divided into 5 groups addressing physical activity, salt intake, fat and oil consumption, fruit and vegetable intake, and regular hypertension medication use. Each group included 4 questions based on the constructs of the Transtheoretical Model of behavior change. For example, for the questions related to regular hypertension medication use, patients were asked: (1) Are you currently taking hypertension medication regularly? Yes/No (2) Do you intend to take hypertension medication regularly in the next 6 months? Yes/No (3) Do you intend to take hypertension medication regularly in the next 1 month? Yes/No (4) Have you taken hypertension medication regularly in the past 6 months? Yes/No.

The scoring method was based on the questionnaire developed by Dr. Nigg and colleagues (13), where the patients’ stage of behavior change was determined by their responses to these four questions. If the response to item 1 was “No,” item 2 was “Yes,” and item 3 was “No,” the individual was considered to be in the contemplation stage. If the response to item 1 was “No” and item 3 was “Yes,” the individual was in the preparation stage. If the response to item 1 was “Yes” and item 4 was “No,” the individual was in the action stage. If the response to item 1 was “Yes” and item 4 was also “Yes,” the individual was in the maintenance stage. The questionnaire section is presented as follows:

(a) *Physical activity:*
  • Do you currently engage in regular physical activity?
  • Do you intend to incorporate regular physical activity into your routine within the next month?
  • Do you intend to incorporate regular physical activity into your routine within the next 6 months?
  • Have you engaged in regular physical activity during the past 6 months?
(b) *Salt intake:*
  • Do you currently consume less than 5 grams of salt per day?
  • Do you intend to consume less than 5 grams of salt per day within the next months?
  • Do you intend to consume less than 5 grams of salt per day within the next 6 months?
  • Have you consumed less than 5 g of salt per day during the past 6 months?
(c) *Oil intake:*
  • Do you currently consume of fatty and fried food?
  • Do you intend to reduce your intake of fatty and fried foods within the next months?
  • Do you intend to reduce your intake of fatty and fried foods within the next 6 months?
  • Have you consumed less fatty and fried food during the past 6 months?
(d) *Fruit and Vegetable intake:*
  • Do you currently consume 5–6 servings of fruits and vegetables per day?
  • Do you intend to consume 5–6 servings of fruits and vegetables daily within the next month?
  • Do you intend to consume 5–6 servings of fruits and vegetables daily within the next 6 months?
  • Have you consumed 5–6 servings of fruits and vegetables daily during the past 6 months?
(e) *Medication:*
  • Do you currently regularly take hypertension medication?
  • Do you intend to take hypertension medication regularly within the next month?
  • Do you intend to take hypertension medication regularly within the next 6 months?
  • Have you taken hypertension medication regularly during the past 6 months?

#### 2.2.1 Face validity

The evaluation process involved both qualitative and quantitative assessments of face validity. Initially, qualitative face validity was assessed through face-to-face interviews with seven healthcare providers, aimed at collecting insights on the appropriateness, difficulty, relevance, and clarity of the subject matter. Subsequently, a quantitative evaluation was conducted with ten nursing students who rated the importance of each question on a 5-point Likert scale, ranging from “very important” (5) to “not important” (1). The impact score for each item was calculated using the formula: Impact score = Frequency (%) × Importance. Here, frequency represents the percentage of respondents rating an item 4 or 5, and items scoring above 1.5 were considered suitable for further analysis. Additionally, content validity was assessed through interviews with 15 experts in health education and nutrition, focusing on grammar, appropriateness, difficulty, relevance, and clarity of the subject matter. The results of the face validity assessment indicated that all items of the tool were deemed appropriate, clear, and important. Furthermore, the quantitative face validity results revealed that all scores exceeded the threshold of 1.5. Based on the recommendations from the 15 experts, some items were revised to enhance qualitative content validity (14).

#### 2.2.2 Content validity

For quantitative content validity, the content validity ratio (CVR) and content validity index (CVI) were calculated based on expert evaluations. Experts rated each item as “not necessary” (1), “useful but not necessary” (2), or “necessary” (3). The CVR was computed using the formula: CVR = [ne−(N/2)]/(N/2), with a minimum acceptable CVR of 0.52 for a panel of 15 experts. The CVI assessed the relevance of scale items, with scores categorized from “not relevant” (1) to “completely relevant” (4). Items with a CVI above 0.79 were deemed acceptable, while those below 0.70 were unacceptable. The scale content validity index (S-CVI) and scale content validity ratio (S-CVR) were derived by averaging the respective CVI and CVR values, with an S-CVI greater than 0.9 considered acceptable. The scale-level content validity ratio (S-CVR) and scale-level content validity index (S-CVI) were calculated to be 0.82 and 0.95, respectively (15, 16).

#### 2.2.3 Reliability

The internal consistency was assessed using the Kuder-Richardson Formula 20 (KR-20), yielding values of 0.75 for physical activity, 0.76 for salt intake, 0.75 for oil intake, 0.77 for fruit and vegetable consumption, and 0.70 for medication adherence. In the test-retest assessment, the questions were administered twice to 30 hypertensive patients who were not part of the study sample. The Intra-class Correlation Coefficient (ICC) was calculated, with a cutoff of 0.70, resulting in values of 0.87, 0.83, 0.86, and 0.84, respectively (17, 18).

### 2.3 Data collection

To ensure coordination among the interviewers and the completion of the questionnaire, a training and orientation session was conducted. In this session, the purpose of each question and the proper way of asking it were explained. Given that the health workers and caregivers participating in this study were responsible for taking the patients’ medical history and measuring their blood pressure, a coordination meeting was also held to standardize and TRAIN them on the proper blood pressure measurement technique, according to the JNC7 guidelines (19).

For the non-adherence intervention group, which included patients in the Precontemplation, Contemplation, and Preparation stages, the following strategies were utilized: In the Precontemplation stage, the intervention aimed to increase patients’ awareness of the importance of blood pressure control and emphasize the benefits of a healthy diet and physical activity. During the Contemplation stage, the intervention focused on assessing the barriers and benefits of behavior change, as well as increasing patients’ awareness of their salt, fatty food, and fruit/vegetable intake levels. In the Preparation stage, the intervention assisted patients in setting achievable goals and taught effective methods to reduce salt and fat intake, as well as increase fruit and vegetable consumption. The perceived pros and cons of changing the behavior were explained in this group.

For the adherence intervention group, which included patients in the Action and Maintenance stages, the intervention provided practical strategies for implementing behavioral changes and enhanced patients’ self-efficacy in executing the changes. Additionally, the intervention supported and encouraged patients to continue positive changes, and taught coping strategies to overcome barriers and challenges.

Since the pre-test questionnaire had already been completed in the previous stage, the educational programs tailored for the non-adherence intervention group consisted of four 45 min sessions over two months. These sessions included lectures, question-and-answer sessions, group discussions, motivational interviews, film screenings, and practical demonstrations with images of patients who had experienced complications due to high blood pressure. Additionally, the experiences of successful patients in controlling their blood pressure were shared during the sessions. At the end of the educational sessions, the materials were provided to the patients in the form of booklets, pamphlets, and self-care cards. For the adherence category, two 45 min educational sessions were held.

Three months after the sessions, the pos*t*-test questionnaires were completed by the researcher for the patients in each of the intervention and control groups, including the adherence and non-adherence categories. At the end of the study and after the questionnaires were completed, the relevant educational sessions were also provided to the control groups using the same methods, such as lectures, question-and-answer sessions, and practical demonstrations.

### 2.4 Statistical analysis

The statistical analyses were conducted using SPSS version 26 software, and a significance level of 0.05 was set. To examine the homogeneity of demographic variables between the intervention and control groups, the Chi-square test and Gamma test were employed.

To compare lifestyle variables, we conducted a within-group analysis to evaluate significant changes in the number of individuals in both the intervention and control groups before and after the intervention, using the McNemar test. For between the group analysis, we conducted either the Fisher's exact test or the Chi-square test, with the choice of the Chi-square test contingent upon whether the expected frequency in each cell was greater than 5. If this criterion was not met, we used Fisher's exact test instead.

The Wilcoxon Signed-Rank Test was employed to assess differences within the control and intervention groups regarding mean systolic blood pressure (SBP) and diastolic blood pressure (DBP) before and after the intervention. Additionally, we applied the Mann–Whitney *U* test to evaluate whether there were statistically significant differences in the means of SBP and DBP between the intervention and control groups, both prior to and following the intervention.

## 3 Results

This study included 120 hypertensive patients from the Nukan Rural Health Center in Kermanshah. The participants were randomly divided into two groups: 60 patients who followed the treatment regimen and 60 patients who did not. The mean age ± SD of the participants in the non-adherence category was 54(±11.80) for the intervention group and 56.97 ± 8.86 for the control group. In the adherence category, the mean ± SD age was 58.77 ± 11.78 for the intervention group and 62.63 ± 13.39 for the control group. Based on the independent *t*-test, there was no statistically significant difference in age between the intervention and control groups for both non-adherence (*p* = 0.275) and adherence to treatment *p* > 0.05 (Table 1).

**Table 1:** Comparison of frequency distribution of demographic characteristics of adherence and non-adherence category.

parameters	Non- adherent category			Adherent category		
	Intervention group number (%)	Control group number (%)	*P*-value	Intervention group number (%)	Control group number (%)	*P*-value
Sex						
Male	10 (55.6)	8 (44.4)	0.573^a^	12 (54.5)	10 (45.5)	0.592^a^
Female	20 (47.6)	22 (52.4)		18 (47.14)	20 (52.6)	
Marital status						
Married	26 (50.0)	26 (50.0)	0.99^a^	29 (51.8)	27 (48.2)	0.301^a^
Single	4 (50.0)	4 (50.0)		1 (25)	3 (75)	
Education level						
Illiterate	15 (62.5)	9 (37.5)	0.114^a^	17 (54.8)	14 (45.2)	0.438^a^
High school	15 (41.7)	21 (58.3)		13 (44.8)	16 (52.2)	
Job						
Housewife	19 (46.3)	22 (53.7)	0.80^a^	17 (45.9)	20 (54.1)	0.379^a^
Worker	2 (66.7)	1 (33.3)		1 (50)	1 (50)	
Free	5 (55.6)	4 (44.4)		5 (62.5)	3 (37.5)	
Unemployed	2 (66.7)	1 (33.3)		0	2 (100)	
Driver	1 (100.0)	0		0	0	
Rancher	0	0		0	1 (100)	
Farmer	0	0		2 (50.0)	2 (50)	
Retired	1 (33.3)	2 (66.7)		5 (83/3)	1 (16.7)	
Income						
No income	9 (52.9)	8 (47.1)	0.22^b^	8 (42.1)	11 (57.9)	0.20^b^
Under one million	17 (53.1)	15 (46.9)		12 (46.2)	14 (53.8)	
One to two million	4 (48.1)	7 (63.6)		10 (66.7)	5 (33.3)	
Smoking						
Yes	5 (62.5)	3 (37.5)	0.448^a^	4 (57.1)	3 (42.9)	0.688^a^
No	25 (48.1)	27 (51.9)		26 (49/1)	27 (50/9)	
Duration of illness						
Less than 2 years	4 (33.3)	8 (66.7)	0.199^b^	7 (53.8)	6 (46.2)	0.187^b^
Between 2 and 5 years	16 (59.3)	11 (40.7)		8 (44.4)	10 (55.6)	
Between 5 and 10 years	4 (40.0)	6 (60)		4(26.7)	11(73.3)	
More than 10 years	6(54.5)	5(45.5)		11(78.6)	3(21.4)	

^a^ Chi-square test.

^b^ Gamma test.

Additionally, other demographic characteristics of the population, including gender, marital status, literacy level, occupation, income level, smoking, and duration of hypertension, did not show a statistically significant difference between the control and intervention groups *p* > 0.05 (Table 1).

Based on the TTM, the goal was to help participants reach the stage of action and maintenance for each of the target behaviors. To assess the effectiveness of the intervention, the study examined the statistical significance of the change in the frequency of participants who transitioned to these two stages (action and maintenance) after the intervention, compared to before the intervention. (Table 2).

**Table 2:** Comparison of frequency of patients in action and maintenance group before and after intervention among non- adherence category and adherence category.

Behaviors	Group	Non-adherent category (60)			Adherent category (60)		
		Before intervention (*n*)	After intervention (*n*)	*p*-value^a^	Before intervention (*n*)	After intervention (*n*)	*p*-value^a^
Physical activity	Intervention	5	14	**0**.**004**	7	12	0.063
	Control	3	3	>0.05	12	13	>0.05
*p*-value^b^		>0.05	**<0.001**		>0.05	>0.05	
Salt intake	intervention	21	29	**0**.**008**	24	27	0.250
	Control	22	22	>0.05	22	23	>0.05
*p*-value^b^		>0.05	**<0.05**		>0.05	>0.05	
Oil intake	Intervention	20	27	**0**.**016**	22	23	>0.05
	Control	18	18	>0.05	19	20	>0.05
*p*-value^b^		>0.05	**<0.05**		>0.05	>0.05	
Fruits and vegetables	Intervention	10	22	**<0.001**	15	18	0.250
	Control	14	14	>0.05	15	15	>0.05
*p*-value^b^		>0.05	**<0.05**		>0.05	>0.05	
Medication	Intervention	0	21	**<0.001**	30	29	>0.05
	Control	0	1	>0.05	30	29	>0.05
*p*-value^b^		>0.05	**<0.001**		>0.05	>0.05	

Bold value indicates the significant difference.

^a^ McNemar test.

^b^ Fisher's exact test and Chi-square test based on expected frequency >5.

Table 2 presents a comparison of the frequency of patients in the action and maintenance stages before and after the intervention, for both the non-adherence and adherence categories. Within group analysis indicated that in the non-adherence category, the McNemar's test showed a significant increase after the intervention in physical activity, limiting salt intake, limiting oil intake, fruit and vegetable consumption, and drug consumption (*p* < 0.05). No significant changes were observed in the control group. In the adherence category, all of the health-related behaviors increased after the intervention, although they did not change significantly. The Fisher exact test and Chi-square test indicated that the between-group analysis revealed no statistically significant differences between the two groups prior to the intervention (*p* > 0.05). However, significant differences emerged following the intervention (*p* < 0.05 or *p* < 0.001). This statistical change was observed exclusively in the non-adherence category, while no significant differences were found in the adherence category (*p* > 0.05).

Systolic blood pressure (SBP) and diastolic blood pressure (DBP) were compared within and between the groups. In within group analysis, in the intervention group, the mean (SD) SBP decreased from 142.88 (±20.87) mmHg to 141.00 (±18.52) mmHg, which was statistically significant (*p* = 0.015). The mean (SD) DBP decreased from 88.17 (±10.30) mmHg to 87.59 (±9.70) mmHg, but this difference was not statistically significant (*p* = 0.154). In the control group, the changes in systolic (*p* = 0.080) and diastolic (*p* = 0.516) blood pressure were not statistically significant. Between-group analysis indicated that no statistically significant differences were observed after the intervention between the intervention and control groups for either SBP or DBP (*p* > 0.05) (Table 3).

**Table 3:** Comparison of systolic and diastolic blood pressure in study groups.

Variable	Group	Non-adherent category			Adherent category		
		Before intervention	After intervention	*p*-value^a^	Before intervention	After intervention	*p*-value^a^
		Mean ± SD	Mean ± SD		Mean ± SD	Mean ± SD	
SBP	Intervention	142.88 ± 20.87	141.00 ± 18.52	**0.015**	119.17 ± 9.92	120.50 ± 9.50	0.083
	Control	142.92 ± 14.20	143.50 ± 14.00	0.080	124.83 ± 13.76	124.75 ± 10.78	0.762
*p*-value^b^		0.55	0.31		0.09	0.13	
DBP	Intervention	88.17 ± 10.30	87.58 ± 9.70	0.154	78.75 ± 8.19	79.17 ± 7.23	0.389
	Control	89.25 ± 8.51	89.08 ± 7.75	0.516	79.83 ± 9.80	80.58 ± 7.62	0.233
*p*-value^b^		0.73	0.51		0.29	0.33	

Bold values indicate the significant difference.

^a^ Wilcoxon signed ranks test.

^b^ Mann–Whitney *U* test.

## 4 Discussion

Participants were categorized into adherent and non-adherent categories based on their medication compliance. It was hypothesized that non-adherent participants were in the early stages of behavioral change specifically, pre-contemplation, contemplation, and preparation regarding physical activity, dietary habits, and medication adherence. Interventions targeting the non-adherence category aimed to raise awareness, address barriers, emphasize benefits, and facilitate goal-setting, thereby promoting progression through the stages of change outlined in the TTM. The primary objective was to inform, motivate, and support non-adherent participants in achieving sustained improvements in health behaviors related to hypertension. For the adherence category, interventions tailored to the action and maintenance stages of the TTM were implemented.

The positive impact of various short-term interventions based on the TTM has been explored in several studies (20–22). The present study found that interventions tailored to the stages of change in the non-adherence category were more effective in promoting a broader range of healthy behaviors compared to the adherence category. A meta-analysis conducted by Imeri et al. [(23)] supports these findings, indicating that the TTM is more effective in facilitating behavioral changes for blood pressure control among individuals with poor adherence than those with moderate adherence (23). Even in the analysis between the two groups, statistically significant differences were observed for the variables of physical activity, salt intake, fat intake, and fruit and vegetable consumption, specifically between the control and intervention groups within the non-adherence category, but not in the adherence category. Furthermore, the findings suggest that patients who adhere to medication are more likely to engage in further lifestyle behaviors. This indicates that lifestyle-based interventions grounded in the staged-matched trans-theoretical model may be more effective for non-adherent individuals, meaning those in the pre-contemplation, contemplation, and action stages.

In the present study, SBP was significantly reduced only in the intervention group of non-adherent patients. However, DBP did not demonstrate a significant change in either the non-adherent or adherent categories. Previous research indicates that effective blood pressure-lowering strategies typically require implementation for more than six months (10). This duration may account for the insufficient impact observed from the tailored interventions based on the TTM in the current investigation.

In accordance with the findings of Chen et al. [(24)], the most significant reduction in blood pressure was observed at the 6-month follow-up following the intervention. This study investigated the effects of a TTM-based intervention on the stages of change, as well as its impact on SBP and DBP. Three months post-training, a significant decrease in diastolic blood pressure was noted, while systolic blood pressure showed no significant change (*β* = −0.802 for DBP and *β* = 1.031 for SBP). At the 6-month follow-up, the intervention significantly reduced both SBP and DBP (*β* = −4.158 for SBP and *β* = −4.580 for DBP). However, at the 12-month follow-up, a similar decrease in blood pressure was observed, although this reduction was not statistically significant (24). In our study, potential reasons for the differential effects on systolic and diastolic blood pressure may include the nature of lifestyle changes, which often have a more immediate impact on systolic pressure (25). Changes observed in mean SBP in patients in this study are similar to those showed previously (26, 27). It appears that short-term interventions for patients with hypertension primarily affect SBP rather than DBP (27). Given that increased markers of oxidative stress have been linked to ventricular function (28) and are noted in hypertensive patients (29), it is proposed that oxidative stress could serve as a potential mediator of blood pressure changes in hypertensive individuals due to exercise and other lifestyle modifications (27). However, to demonstrate results for reducing SBP below the clinical recommendation threshold of 140 mmHg, longer-term lifestyle changes, such as exercise, are necessary beyond the duration of our study. In addition, the limited follow-up period of three months may be the reason for the non-significant differences observed in the between-group analysis, as it might not have allowed sufficient time for the intervention to produce observable changes in blood pressure between the two groups. Further investigation into this phenomenon is warranted.

In the present study, a statistically significant transition to the action and maintenance stages was observed three months post-intervention among the non-adherence intervention group. This transition was evident across all physical activity behaviors, as well as in salt and oil intake, fruit and vegetable consumption, and medication adherence. Notably, the most pronounced improvements within the non-adherent group were found in medication adherence, followed closely by enhancements in fruit and vegetable consumption.

Similarly, in the previously mentioned study by Chen et al., the stages of behavior change in the intervention group were statistically significant at 3, 6, and 12 months post-intervention *p* < 0.05 (24). Furthermore, a systematic review and meta-analysis indicated that verbal educational interventions had a small but significant positive effect on medication adherence among hypertensive patients (30).

A review indicates that the TTM serves as a more sensitive measure for assessing progress in dietary intake changes. Even in instances where actual food consumption does not exhibit significant alterations, positive outcomes may still be evident, such as increases in an individual's decisional balance or self-efficacy (the belief in one's ability to perform a behavior) or advancement through the various stages of behavioral change delineated in the TTM (31). Vergeld et al. [(32)] found that changes in self-efficacy, outcome expectancies, and coping planning were associated with stage progression for fruit and vegetable intake, although this was not the case for physical activity. Their study collectively examined the stages of behavioral change and their impact on health-related behaviors (32). Conversely, our study reported a positive effect on physical activity behavior following the intervention, despite the fact that physical activity had the lowest number of participants progressing to the subsequent stages compared to other healthy behaviors assessed.

The application of intervention strategies aimed at promoting physical activity to improve blood pressure aligns with previous studies utilizing the TTM. For instance, Ghaffari's study demonstrated that following the implementation of interventions, over 50% of participants progressed to the fourth and fifth stages of the model (33). In the context of physical activity promotion, TTM-based interactive multimedia software has proven effective in facilitating individuals’ advancement through the stages of change, significantly enhancing physical activity levels, which may contribute to hypertension prevention (34). The TTM is particularly suitable for improving physical activity, as engaging in physical activities involves cognitive and mental stages. Following the initiation of the desired behavior, individuals may stabilize (maintain) their activity levels or, conversely, regress to a state of inactivity (22).

## 5 Strength and limitations

A major strength of this study is the use of the TTM to tailor the interventions to the specific needs and readiness of the participants. By targeting the non-adherent category with strategies to promote progression through the stages of change, the intervention was able to achieve significant improvements in a range of healthy behaviors, including physical activity, dietary modifications, and medication adherence. There were no missing data, ensuring that our analysis remained robust and valid under the assumptions of the statistical tests applied. Moreover, the significant reduction in systolic blood pressure observed in the non-adherent intervention group underscores the potential clinical benefits of this approach.

One of the key limitations of this study is the relatively short follow-up period. The current study may have been limited by the duration of the interventions, which may not have been sufficient to fully observe the impact on diastolic blood pressure and the maintenance of other healthy behaviors. In real-world scenarios, lifestyle changes typically require more than six months to stabilize, as suggested by the Transtheoretical Model (TTM). Thus, it is possible that participants may not have reached the maintenance phase during this brief follow-up. Consequently, the internal validity of our study may not be very robust. Another limitation pertains to our categorization of stages of change, which was based solely on medication use rather than encompassing related behaviors, such as physical activity. As evidence shows, other aspects of lifestyle may affect patients' adherence to healthy behaviors (35). Results may restrict the generalizability of our findings to patients in different geographic and cultural contexts, warranting caution in the interpretation of the results. Additionally, the study relied on self-reported measures of adherence and behavior change, which can be subject to social desirability bias and recall bias. Another limitation is that the study did not consider stress management as part of the intervention strategies, which is an important factor in the management of hypertension. Additionally, some of the medications prescribed to the participants may not have been based on the latest evidence-based guidelines for hypertension management, which could have influenced the study outcomes.

## 6 Conclusion

The study indicates that tailored interventions based on the TTM are effective for non-adherent hypertensive patients in the early stages of behavior change. These interventions emphasize increasing awareness, addressing barriers, and facilitating goal-setting to promote progression to the action and maintenance stages for health behaviors, including physical activity, dietary changes, and medication adherence. Although the study did not demonstrate lasting effects on diastolic blood pressure and showed limited improvements among adherent patients, it underscores the TTM's potential to enhance adherence and clinical outcomes for individuals with hypertension. These findings highlight the significance of personalized interventions grounded in the TTM framework to achieve better health outcomes for hypertensive individuals.

## Data Availability

The original contributions presented in the study are included in the article, further inquiries can be directed to the corresponding author.
